# Surgical quadriceps lengthening can reduce quadriceps spasticity in chronic stroke patients. A case-control study

**DOI:** 10.3389/fneur.2022.980692

**Published:** 2022-10-13

**Authors:** Andrea Merlo, Martina Galletti, Paolo Zerbinati, Paolo Prati, Francesca Mascioli, Giacomo Basini, Chiara Rambelli, Stefano Masiero, Davide Mazzoli

**Affiliations:** ^1^Gait and Motion Analysis Laboratory, Sol et Salus Hospital, Rimini, Italy; ^2^Neuro-Orthopedic Unit, Sol et Salus Hospital, Rimini, Italy; ^3^Section of Rehabilitation, Department of Neuroscience, University of Padova, Padua, Italy

**Keywords:** stroke, spasticity, surgery, aponeurectomy, Modified Tardieu Scale

## Abstract

**Background:**

Muscle overactivity is one of the positive signs of upper motor neuron lesions. In these patients, the loss of muscle length and extensibility resulting from soft tissue rearrangement has been suggested as a contributing cause of muscle overactivity in response to stretching.

**Objective:**

To assess the effects of surgical lengthening of the quadriceps femoris (QF) muscle-tendon unit by aponeurectomy on muscle spasticity.

**Methods:**

This is a case-control study on chronic stroke patients with hemiparesis that have undergone lower limb functional surgery over a 8-year period. CASEs underwent corrective surgery for both the foot and knee deviations, inclusive of a QF aponeurectomy. Controls (CTRLs) underwent corrective surgery for foot deviations only. QF spasticity was assessed with the Modified Tardieu Scale (MTS) before and 1 month after surgery. The Wilcoxon test was used to assess MTS variations over time and the Mann–Whitney test was used to verify the presence of group differences at the 1 month mark.

**Results:**

Ninety-three patients were included: 57 cases (30F, 1–34 years from lesion) and 36 controls (12F, 1–35 years from lesion). Before surgery, both CASEs and CTRLs had similar MTS scores (median MTS = 3) and functional characteristics. One month after surgery, QF spasticity was significantly lower in the CASEs compared to CTRLs (*p* = 0.033) due to a significant reduction of the median MTS score from 3 to 0 in the CASE group (*p* < 0.001) and no variations in the CTRL group (*p* = 0.468). About half of the cases attained clinically significant MTS reductions and complete symptom relief even many years from the stroke.

**Conclusions:**

Functional surgery inclusive of QF aponeurectomy can be effective in reducing or suppressing spasticity in chronic stroke patients. This is possibly a result of the reduction in neuromuscular spindle activation due to a decrease in muscle shortening, passive tension, and stiffness.

## Introduction

In patients with upper motor neuron lesions (UMNL), weakness consequent to paresis leaves the affected muscles immobilized. The prolonged maintenance in the shortened position produces changes in soft tissues, with a progressive modifications of the muscle rheologic properties (1), leading to muscle contracture (2). UMNL and muscle contracture are the cause of a form of muscle overactivity that is often referred to as non-reflex hypertonia, intrinsic hypertonia (3, 4), or spastic dystonia (5–7). Regardless of the trigger—pain, gravity, etc.—this muscle overactivity can be present in completely flaccid muscles and can last for several hours a day (8, 9) resulting in an even further shortening, thus aggravating muscle contracture, in a vicious cycle (2).

Muscle immobilization also impairs post-activation depression, which is key in the progressive development of spasticity (3), and one of the positive signs occurring after UMNL. This is characterized by an abnormal reflex muscle activation in response to a fast stretch, at rest, and by impaired derecruitment (3, 5, 6, 10). More in general, hyperexcitability of the stretch reflex produces spasticity, clonus, and the increase of deep tendon reflexes (3).

The link between these two different types of muscle overactivity has been highlighted in recent literature (3, 5, 6). The reduced extensibility—i.e., the muscle's ability to absorb part of the applied stretch through its own elongation—because of muscle shortening, increased stiffness and increased internal viscosity (2, 11), might cause “any pulling force to be transmitted more readily to the spindles,” thus increasing spasticity (2, 3, 12).

Following this reasoning, a recovery in muscle length and ability to elongate should result in a reduction of spindle activation during muscle stretch, and should in turn result in a reduction of spasticity. We hypothesized that surgical muscle lengthening should result in reduced spasticity at least in those patients whose hyperreflexia is determined by excessive spindles activity (3).

Surgical muscle lengthening is a common procedure used with neurological patients during neuro-orthopedic surgery in order to correct joint deviations (13–15). Two recent and independent studies on children affected by cerebral palsy (CP) have reported a significant decrease in muscle spasticity after muscle lengthening surgery (16, 17). However, interesting these findings may be, we still have no comparable results reported in adult stroke survivors. Only one preliminary report, seems to confirm this hypothesis (18). Nevertheless, this was a single-arm study with a limited sample size. A case-control study would be the appropriate design in order to test whether surgical muscle lengthening of a target muscle group, performed with a standardized procedure, could lead to a decrease in spasticity.

In this case-control study, we compared the effects of lower limb functional surgery on quadriceps femoris (QF) spasticity in two parallel groups of stroke patients who underwent surgery including or not including QF aponeurectomy.

## Methods

### Study design and settings

In this observational case-control study, we retrospectively analyzed data from stroke patients with chronic hemiplegia who had undergone lower limb functional surgery at our institution over the period of time between November 2012 and November 2020. Patients were evaluated before and 1 month after surgery.

### Inclusion and exclusion criteria

We included adult patients with the following criteria: (1) left or right hemiparesis consequent to an ischemic or hemorrhagic stroke; (2) chronic stroke; (3) first functional surgery to correct lower limb deviations; (4) available clinical Modified Tardieu Scale (MTS) score at QF both before and 1 month after surgery; (5) availability of a signed informed consent. The exclusion criteria were: (1) previous neurotomies to correct lower limb deformities, (2) previous surgery on the lower limbs, and (3) treatment with botulinum toxin up to 6 months prior to our evaluation.

This study was approved by the Local Ethics Committee (CEIIAV Prot. 5953/2017 and 7166/2020).

### Grouping

According to the aim of this study, patients were divided into two groups, based on the presence/absence of QF surgical lengthening by aponeurectomy, as follows.

#### CASE group

Patients who underwent functional surgery to correct both foot and knee deviations—including surgical lengthening of the triceps surae muscle-tendon unit and foot correction in the frontal plane when necessary—and QF aponeurectomy, without other interventions on the thigh muscles (e.g., hamstring).

#### CTRL group

Patients who underwent distal functional surgery to correct foot deviations only.

QF aponeurectomy was the only difference in treatment between CASEs and CTRLs.

For each patient, the surgical procedure was decided based on both clinical and instrumental gait assessment with gait analysis and dynamic EMG (11, 19). The complete description of the surgical procedures is provided in Supplementary Data. One surgeon (author PZ) performed all surgeries.

### Primary outcome

The primary outcome of the study was QF spasticity, assessed with the score of the MTS. This is a semi-quantitative, 5-level ordinal scoring system based on the strength and duration of the stretch reflex (20). The following MTS formulation was used (20):

0—Absence of muscle reaction.

1—Weak resistance throughout the stretching movement, without a clear catch.

2—A clear catch, followed by release.

3—Fatiguing clonus, < 10 s.

4—Non fatiguing clonus, >10 s.

Muscle reaction was measured during passive stretching of the knee extensors at V2 (21), with the patient lying supine on a bed, with the affected hip joint flexed at 30° and the contralateral limb extended. All the participants' measurements were performed by the same two experienced examiners.

### Clinical variables

Demographic data (age, sex), the patients' history including stroke type, years from lesion, affected side, clinical assessments including joint range of motions (ROMs), strength using the Manual Muscle Test (MMT), spasticity using the MTS, and functional assessments of walking using the Functional Ambulation Category (FAC) and the Rivermead Mobility Index (RMI) were retrieved from the laboratory database. These were used to characterize the sample and to allow for groups comparison at baseline.

### Statistical analysis

The comparison between baseline characteristics of CASEs and CTRLs was carried out using the *t*-test for numerical variables, the Mann–Whitney *U*-test for ordinal variables and with the Fisher's test for dichotomous variables.

The Wilcoxon test was used to analyze MTS variations with respect to baseline values in the two groups. The Mann–Whitney test was used to verify the presence of group differences at the 1 month mark. The presence of an association between a post-operative spasticity (absent when MTS = 0, and present otherwise) and the number of years since the lesion was assessed with the *t*-test.

Along with average results, variations at the single subject level were analyzed. The number of patients who either showed an Improvement (I), were Stable (S), or showed Worsening (W) in the MTS score at the follow-up assessment was determined for both groups (22). A reduction in the MTS score ≥1 point was used to classify a patient in group I. Similarly, an increase >1 was required to classify a patient in group W. Patients were otherwise classified in group S. This allowed to fulfill a 2 × 3 contingency table that summarized the variations (W, S, I) for the two groups (CASE and CTRL). The Freeman–Halton extension of the Fisher's Exact test for a 2 × 3 contingency table was used to analyze the difference in proportions of W, S, and I patients between CASEs and CTRLs (22).

Statistical significance was set at 5% for all analyses. Data analysis was performed using Jamovi (version 1.6.23).

### Power analysis

The Mann-Whitney test requires a sample of 106 patients to achieve an 80% power in the occurrence of data with normal distribution, an allocation ratio of 1, and a medium effect size (*d* = 0.5). Our data present a peaked distribution (kurtosis > 0, modal value = 3) and allocation ratio of about 1.5. In this case, a sample of 70 patients would warrant achieving an 80% power when rejecting the null hypothesis of no difference between groups in the presence of medium effect size (*d* = 0.5) (23).

### Reporting guidelines

The manuscript has been written according to the Strobe checklist for case-control studies.

## Results

A total of 93 patients met the inclusion criteria: 42 women and 51 men, with a mean age of 55 (11) years, at 4.2 (2.2) years post lesion. Of these, 57 belonged to the CASE group and 36 to the CTRL group. Demographic and clinical characteristics of both CASE and CTRL groups are reported in Table 1.

**Table 1:** Sample characteristics for both CASEs and CTRLs.

	**CASEs**	**CTRLs**
Sample size	57	36
Gender (F/M)	30/27	12/24
Age–years	53.8 (11.5)	56.5 (10.7)
Affected side (Left/Right)	35/22	19/17
Type of stroke (haemorrhagic/ischemic)	26/31	11/25
Time from stroke, years	5.94 (6.25); 0.8–34.5	5.6 (6.22); 1.2–35.2
FAC	4; 1–5	4; 1–5
RMI	12; 3–15	10; 1–14
MTS Score at the QF	3; 0–4	3; 0–3
Duncan-Ely	1; 0–4	1; 0–4
Hip flexors MRC	4-; 1–5	3; 1–5
Quadriceps MRC	5; 2–5	4; 2–5
Passive knee flexion (degrees)	128 (12.5); 90–130	123 (13.1); 70–130
Passive knee extension (degrees)	1.5 (4.0); 0–15	2.3 (4.5); 0–15
Previous BoNT at the quadriceps (yes/no/n.a.)	46/8/3	31/3/2
Use of walking aids (with/without/n.a.)	35/21/1	26/10/0
Distal surgical procedures
TS lengthening	57	36
FDL-FHL tenotomy	55	34
SPLATT	27	16
Other tendon transfers	18	19
Proximal surgical procedures
QF lengthening by aponeurectomy	57	0

Data are presented as count, mean (standard deviation) and range for numerical variables and as median and range for ordinal variables.

FAC, Functional ambulatory category; RMI, Rivermead mobility index; MTS, Modified tardieu scale; QF, Quadriceps femoris; MRC, Medical research council; BoNT, Botulinum NeuroToxin; n.a., not available; TS, Triceps surae; FDL, Flexor digitorum longus; FHL, Flexor hallucis longus; SPLATT, Split tibialis anterior tendon transfer.

For all the reported variables, no significant group differences were found at the baseline evaluation. At baseline assessment the two groups presented the same level of QF spasticity at the MTS score.

### Primary outcome

**Figure 1:**
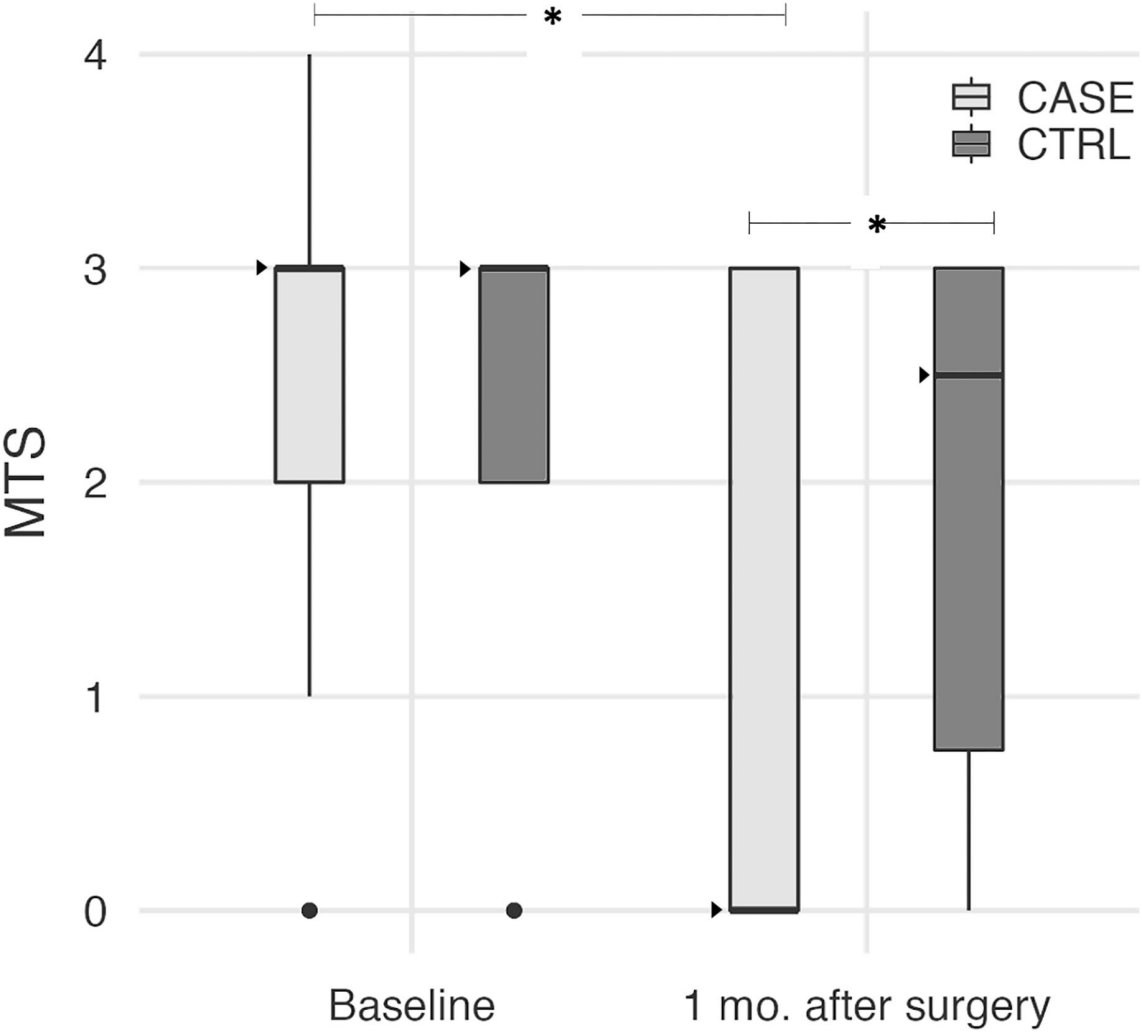
Boxplot diagrams of the MTS score at baseline and 1 month after surgery in both CASEs and CTRLs. Median values are shown as dark triangles. MTS significantly decreased in the CASE group only (Wilcoxon test, *p* < 0.001), with the median score reduced from 3 to 0. After surgery, MTS became significantly lower in CASEs compared to CTRLs (Mann-Whitney test, *p* = 0.033). Asterisks indicate these statistically significant differences.

One month after surgery, QF spasticity as assessed by MTS was significantly lower in CASEs than in CTRLs (Mann-Whitney test *p* = 0.033). This was due to a significant reduction of the median MTS score from 3 to 0 in the CASE group (Wilcoxon test, *p* < 0.001), while it did not vary from the baseline level in the CTRL group (Wilcoxon test, *p* = 0.468), as shown in Figure 1.

A two-way table with MTS values before and after surgery is presented in Table 2 for the CASE group. It is worth mentioning that 26/57 CASE subjects were completely relieved from QF spasticity as assessed by MTS at the 1 month mark, with MTS decreasing from 3 to 0 in 17 cases, from 2 to 0 in 7 cases and from 1 to 0 in 2 cases. The two-way table for the CTRL group is presented in Table 3.

**Table 2:** Two-way table of MTS scores in the CASE group (*N* = 57) at baseline and 1 month after surgery inclusive of QF aponeurectomy.

	**MTS at the quadriceps 1 month after QF aponeurectomy**			
**MTS at the quadriceps at baseline**	**0** **(*N* = 30)**	**1** **(*N* = 0)**	**2** **(*N* = 4)**	**3** **(*N* = 23)**
0 (*N* = 5)	4	0	0	1
1 (*N* = 2)	2	0	0	0
2 (*N* = 10)	7	0	2	1
3 (*N* = 38)	17	0	2	19
4 (*N* = 2)	0	0	0	2

**Table 3:** Two-way table of MTS scores in the CTRL group (*N* = 36) at baseline and 1 month after surgery inclusive of QF aponeurectomy.

	**MTS at the quadriceps 1 month after** **QF aponeurectomy**			
**MTS at the quadriceps at baseline**	**0** **(*N* = 9)**	**1** **(*N* = 1)**	**2** **(*N* = 8)**	**3** **(*N* = 18)**
0 (*N* = 7)	5	0	0	2
1 (*N* = 0)	0	0	0	0
2 (*N* = 10)	1	1	7	1
3 (*N* = 19)	3	0	1	15

When considering a Minimal Clinical Important Difference (MCID) of 1 in the MTS variation for all subjects, the following 2 × 3 contingency table was obtained (Table 4). After surgery, about 52% (95% CI: 39–66%) of the CASEs had a reduction in spasticity, while this happened in only 17% (95% CI: 6–33%) only of the CTRLs.

**Table 4:** MTS variation 1 month after surgery in CASEs and CTRLs.

	**Improved**	**Stable**	**Worsened**	**Total**
**CASE**	30	25	2	57
**CTRL**	6	27	3	36
**Total**	36	52	5	93

MCID of 1 was used in the assessment of spasticity variation by MTS. The null hypothesis of equivalence between CASEs and CTRLs was rejected (*p* = 0.001).

Even with this approach, the equivalence between CASEs and CTRLs at the 1 month mark can be rejected (*p* = 0.001). A similar result was obtained when considering a MCID of 2 (Table 5), with 24 improved patients and 32 stable patients in the CASE group and 4 improved patients and 30 stable patients in the CTRL group (*p* = 0.002, Fisher's Exact test). Only one patient from the CASE group worsened after surgery, along with two patients from the CRTL group.

**Table 5:** MTS variation 1 month after surgery in CASEs and CTRLs.

	**Improved**	**Stable**	**Worsened**	**Total**
**CASE**	24	32	1	57
**CTRL**	4	30	2	36
**Total**	28	62	3	93

MCID of 2 was used in the assessment of spasticity variation by MTS. The null hypothesis of equivalence between CASEs and CTRLs was rejected (*p* = 0.002).

This suppression of spasticity (yes/no) after QF aponeurectomy was not related to the amount of time following the stroke (*p* = 0.437, *t*-test), with MTS score decreasing from 3 to 0 in patients both 1 and 22 years after the stroke.

## Discussion

This study presents, for the first time, the short-term effect of QF lengthening by aponeurectomy on QF spasticity in a sample of adult stroke patients.

The main finding is that spasticity was significantly reduced in the sample of patients who underwent QF aponeurectomy but not in controls, with a median reduction of 3 points at the MTS and a significant between-group difference at the 1 month mark. To the best of our knowledge, there are no available studies in literature discussing hemiparetic adult subjects that show relief in spasticity after surgical muscle lengthening. In CP children only, two studies have found similar results on muscle tone reduction assessed by the Modified Ashworth Scale (MAS) after neuro-orthopedic surgery on the lower limbs (16, 17). Deltombe et al. reported a reduction in the MAS score after surgery in an adult stroke sample (24). In their study, however, 12 out of 18 patients had selective tibial nerve neurectomy, so reduction in muscle tone was clearly expected.

A further finding of our study is that QF lengthening by aponeurectomy had a large effect on approximately half of the CASEs, with MTS dropping from 2 and 3 to 0, and no effect on the remaining half of the CASEs (see Tables 2, 3).

Based on this result, we hypothesize that in chronic stroke patients two different phenomena might exist that contribute to exaggerated stretch reflexes, one of which is reduced or abolished by aponeurectomy. Along with the well-known central phenomenon (2, 24), a peripheral cause might be the trigger, linked to the loss of muscle length and extensibility (5). The release of the distal part of both muscle and muscular fascia consequent to aponeurectomy determines a reduction in the overall muscle passive tension and stiffness, thus resulting in an improved tolerance to stretching. This should lead to both a reduction and a temporal delay of the force transmitted to spindles during the stretching maneuver (2, 3). The reduction in spindle stimulation may result in a decrease in the peripheral inputs that trigger the stretch reflex (6).

A review by Naro et al. (25) points to the overall effectiveness in the reduction of spasticity of other non-pharmacological interventions, which target a reduction in the viscoelastic properties of the connective tissue (e.g., stretching, dry needling, extracorporeal shock-wave therapy, and ultrasound therapy). However, more research is needed to build clear evidence (26). In particular, high-dose stretching applied on muscles in stroke patients has reduced spasticity as measured by MTS or MAS (27, 28) as well as muscle stiffness and viscosity (29). Similarly, physiotherapist-delivered dry needling reduced spasticity as assessed by MAS as well as resistance to passive mobilization in paretic muscle and muscle structure as assessed by ultrasound (30, 31). These findings support our hypothesis that surgical lengthening may change the muscle ability to encode afferent information. The reduction in the pulling force delivered to muscle spindles, which stand on a reduced tension position at rest, could result in a lower response to muscle stretching. Since this hypothesis is new in literature, it deserves further investigation by properly designed prospective studies (e.g., RCTs) on large samples, assessing different muscle groups, and inclusive of neurophysiological assessments (e.g., H/M ratio) as well as long-term follow-ups. These may promote a better understanding and treatment of post-stroke spasticity. In addition, preventing muscle shortening in the acute phase after stroke warrants special attention from a rehabilitative point of view (5, 8).

A further mechanism that may play a role in the reduction of QF spasticity during the Tardieu maneuver, following multilevel functional surgery, is the modification in the heteronomous reflexes. The presence of pathways projecting from the soleus to the quadriceps have been found in healthy humans (32, 33). The contribution of intersegmental facilitative pathways linking the quadriceps to the soleus on the paretic side in stroke individuals has been described in literature (34). Our study design, where CTRLs underwent triceps surae lengthening, allows for the exclusion of this hypothesis. The inclusion of a CTRL group allowed us to check that the reduced QF spasticity was not favored nor determined by a potential reduction in triceps spasticity after surgical muscle lengthening. This result is in line with current literature. When the triceps surae spasticity has been treated by selective neurotomy, as in a study by Deltombe et al. no effects were found in either the quadriceps or the hamstring (35).

Focusing on the CASE group, spasticity improved at the MTS score in half of the sample, while the other half remained stable. Such sharp division made us question about the existence of different underlying phenomena that may result in the same clinical patterns. Patients whose stretch reflex was reduced after QF aponeurectomy may have had a type of overactivity mostly related to the shortening of peripheral structures. This would also lead to the shortening of neuromuscular spindles, and therefore to an increased response to stretching. On the other hand, patients who did not improve after surgery might have had exaggerated stretch reflexes mainly related to central mechanisms. These patients may not be as sensitive to periphery-targeted interventions such as QF aponeurectomy, as the first half of the sample was. Moreover, some of these patients might have had a combination of both, thus increasing the complexity of clinical manifestations.

It is worth remembering that this was the first study highlighting the effects of QF lengthening surgery on muscle stretch reflex. New studies are necessary to keep investigating on this topic and its underlying hypotheses. Future studies should focus on neurophysiological measures to differentiate the peripheral and the reflex components of muscle overactivity after stroke, e.g., by studying the H/M reflex (36, 37) and by assessing the Tonic Stretch Reflex Threshold (38, 39). If confirmed, our results would be of clinical relevance and would help clinicians in the choice of the most appropriate and tailored approach.

From the clinical point of view, muscle lengthening by aponeurectomy has several advantages. It is a simple, minimally invasive procedure that does not require highly specialized surgeons. It guarantees an immediate recovery of joint ROM even in stroke patients several years after the acute event. In subacute patients it could ensure an immediate recovery of joint ROM. In the long term, this translates to an improvement in the quality of life, and prevents the establishment of chronic soft tissue retractions that amplify the vicious cycle between muscle shortening and muscle overactivity that is key in the development of acquired deformities in neurological patients. We therefore suggest surgical muscle lengthening to become part of the usual management of spasticity, also in patients already in the subacute phase.

### Sample characteristics and external validity of the results

In this study, we enrolled chronic patients who sustained a stroke (time from stroke 5.8 ± 6.2 years; range 0.8–35.2 years) with hemiparesis. On the one hand, the inclusion of patients selected for functional surgery, may represent a selection bias, and this must be taken into account. On the other hand, acquired lower limb deviations are a frequent outcome in stroke survivors, where muscle shortening is developed over the course of many years. This is one of the main causes for treatment, especially after many years from the initial lesion.

All patients in the sample had a clinical history of muscle overactivity and/or soft tissue contracture of the lower limbs, a walking pattern characterized by stiff knee gait (SKG) and equinus foot deformity, and a suitable level of functional walking (FAC median score 4 both in CASEs and CTRLs; RMI median score 10–12). In the patient sample, SKG may result from several combinations of its causes, including a lack of push-off due to ankle joint deviation, a possible braking activity of an overactive quadriceps and/or a possible weakness of hip flexors (40–44). These characteristics are common for chronic post-stroke patients with acquired lower limb deformities typically referred to rehabilitation services. This supports the external validity of our results in this kind of chronic stroke patient.

### Study limitations

It would be fitting to collect data from patients in the long-term, to know whether the effects of surgery still persist, and for how long. Patients referred to our Institute usually come from all over the country, and it is often very challenging to have them come back for follow-up evaluations. This lack of long-term results represents the main limitation in our study, which mainly focuses on the short-term effects of QF aponeurectomy. An early assessment, at the 1 month mark, has allowed us to observe the effect of surgical lengthening on spasticity. However, to understand the clinical impact of this procedure on both the patients' function and quality of life, it is necessary to track the evolution of spasticity during the following months, alongside all the factors with which it can interact.

In addition, the inclusion of patients only treated with functional surgery—albeit necessary—exposes the study to a clear selection bias, as discussed above.

Finally, the use of a sole clinical scale to assess spasticity may not be completely reliable and neurophysiological measures could be used to better investigate the effect of surgical muscle lengthening.

## Conclusions

Our results show that functional surgery inclusive of QF aponeurectomy can be effective in reducing or suppressing QF spasticity 1 month after surgery, both in patients 1 year and at 22 years after the stroke. This is possibly a consequence of the reduction in spindle activation due to a decrease in muscle shortening, passive tension and stiffness. These results suggest that surgical muscle lengthening can be a viable option in the management of spasticity in stroke patients.

## Data availability statement

The raw data supporting the conclusions of this article will be made available by the authors, without undue reservation.

## Ethics statement

The studies involving human participants were reviewed and approved by CEIIAV Prot. 5953/2017 and 7166/2020. The patients/participants provided their written informed consent to participate in this study.

## Author contributions

AM and MG: conceptualization and formal analysis. PZ, PP, FM, GB, CR, DM, and AM: data curation. AM: methodology. DM: supervision. AM, MG, GB, and CR: writing—original draft. AM, SM, and DM: writing—review and editing. All authors contributed to the article and approved the submitted version.

## Funding

This study was entirely funded by our Institution.

## Conflict of interest

The authors declare that the research was conducted in the absence of any commercial or financial relationships that could be construed as a potential conflict of interest.

## Publisher's note

All claims expressed in this article are solely those of the authors and do not necessarily represent those of their affiliated organizations, or those of the publisher, the editors and the reviewers. Any product that may be evaluated in this article, or claim that may be made by its manufacturer, is not guaranteed or endorsed by the publisher.

## Abbreviations

CP: Cerebral Palsy
FAC: Functional Ambulation Category
MAS: Modified Ashworth Scale
MCID: Minimal Clinical Important Difference
MMT: Manual Muscle Test
MTS: Modified Tardieu Scale
RMI: Rivermead Mobility Index
ROM: Range of Motion
QF: Quadriceps Femoris
SKG: Stiff Knee Gait
UMNL: Upper Motor Neuron Lesion.
