# Supplementary material for: Surgical quadriceps lengthening can reduce quadriceps spasticity in chronic stroke patients. A case-control study
Source: Front Neurol. 2022 Oct 13;13:980692. doi: 10.3389/fneur.2022.980692 (PMC9606420; doi:10.3389/fneur.2022.980692)
Supplement: Supplementary file 1 [file Table_1.DOCX]

Supplementary Material

Surgical Quadriceps Lengthening Reduces Quadriceps Spasticity in Chronic Stroke Patients. A Case-Control Study

Andrea Merlo^1^, Martina Galletti^1,*^, Paolo Zerbinati^2^, Paolo Prati^1^, Francesca Mascioli^1^, Giacomo Basini^1^, Chiara Rambelli^1^, Stefano Masiero^3^, Davide Mazzoli^1^

# Supplementary Data

## Functional surgery and post-surgical rehabilitation protocol

The equinus foot correction performed by the surgeon involved either the lengthening of the Achilles tendon (Hoke’s procedure) or the lengthening of the gastro-soleus complex (Vulpius procedure). The correction of supination and of the varus component, when necessary, was achieved by a tuned combination of the following procedures: posterior tibial tendon lengthening; release of toe flexor tendons; split anterior tibialis transfer; anterior transfer of the flexor hallux longus; posterior transfer of the flexor digitorum longus, extensor hallucis longus (EHL) transfer on the fourth metatarsal bone.

During QF aponeurectomy, the distal quadriceps muscle was exposed, the rectus femoris muscle was carefully freed from the other quadriceps muscles and subsequently lengthened at the myotendinous junction. The vastus intermedius muscle was then identified beneath the rectus femoris muscle and lengthened by transecting the muscle aponeurosis over the muscle belly. When necessary, other proximal interventions were performed in conjunction with QF aponeurectomy, such as percutaneous tenotomy of the medial and/or lateral knee flexors, hamstring lengthening, and/or tenotomy of the long adductor muscle.

After surgery, all patients received a standardized intensive rehabilitation treatment. The rehabilitation protocol performed after surgery consisted of 24 sessions lasting 90 min, 6 days a week for a 4-week period. It included passive and active ankle mobilization, resistance and stretching exercises, and early gait training with a non-articulated ankle foot orthosis.
